# Impact of the Allowed Compositional Range of Additively Manufactured 316L Stainless Steel on Processability and Material Properties

**DOI:** 10.3390/ma14154074

**Published:** 2021-07-22

**Authors:** Felix Großwendt, Louis Becker, Arne Röttger, Abootorab Baqerzadeh Chehreh, Anna Luise Strauch, Volker Uhlenwinkel, Jonathan Lentz, Frank Walther, Rainer Fechte-Heinen, Sebastian Weber, Werner Theisen

**Affiliations:** 1 Chair of Materials Technology, Ruhr-University Bochum, 44801 Bochum, Germany; louis.becker@ruhr-uni-bochum.de (L.B.); lentz@wtech.rub.de (J.L.); weber@wtech.rub.de (S.W.); theisen@wtech.rub.de (W.T.); 2 Chair of New Manufacturing Technologies and Materials, University of Wuppertal, 42651 Solingen, Germany; roettger@uni-wuppertal.de; 3 Department of Materials Test Engineering, Technical University Dortmund, 44227 Dortmund, Germany; abootorab.chehreh@tu-dortmund.de (A.B.C.); frank.walther@tu-dortmund.de (F.W.); 4 Leibniz Institute for Materials Engineering—IWT, 28359 Bremen, Germany; strauch@iwt-bremen.de (A.L.S.); uhl@iwt-bremen.de (V.U.); fechte@iwt-bremen.de (R.F.-H.); 5 MAPEX Center for Materials and Processes, University of Bremen, 28359 Bremen, Germany

**Keywords:** additive manufacturing, powder bed fusion-laser beam/metal (PBF-LB/M), stainless steel (316L), microstructure, solid-state cracking

## Abstract

This work aims to show the impact of the allowed chemical composition range of AISI 316L stainless steel on its processability in additive manufacturing and on the resulting part properties. ASTM A276 allows the chromium and nickel contents in 316L stainless steel to be set between 16 and 18 mass%, respectively, 10 and 14 mass%. Nevertheless, the allowed compositional range impacts the microstructure formation in additive manufacturing and thus the properties of the manufactured components. Therefore, this influence is analyzed using three different starting powders. Two starting powders are laboratory alloys, one containing the maximum allowed chromium content and the other one containing the maximum nickel content. The third material is a commercial powder with the chemical composition set in the middle ground of the allowed compositional range. The materials were processed by laser-based powder bed fusion (PBF-LB/M). The powder characteristics, the microstructure and defect formation, the corrosion resistance, and the mechanical properties were investigated as a function of the chemical composition of the powders used. As a main result, solid-state cracking could be observed in samples additively manufactured from the starting powder containing the maximum nickel content. This is related to a fully austenitic solidification, which occurs because of the low chromium to nickel equivalent ratio. These cracks reduce the corrosion resistance as well as the elongation at fracture of the additively manufactured material that possesses a low chromium to nickel equivalent ratio of 1.0. A limitation of the nickel equivalent of the 316L type steel is suggested for PBF-LB/M production. Based on the knowledge obtained, a more detailed specification of the chemical composition of the type 316L stainless steel is recommended so that this steel can be PBF-LB/M processed to defect-free components with the desired mechanical and chemical properties.

## 1. Introduction

In times of climate change and the steadily increasing scarcity of resources, special requirements are placed on the production of components concerning sustainability [1]. This is one of the reasons why interest in Additive Manufacturing (AM) processes has grown steadily in recent decades [2]. Due to the layer-by-layer built-up, a resource-efficient production of highly complex, net-shaped components is possible, enabling the high potential for lightweight construction to be fulfilled. Furthermore, it allows the time-efficient production of highly customized components without using product-specific tools [3]. An AM process used in particular for metal processing is the Powder Bed Fusion-Laser Beam/Metal (PBF-LB/M) process, in which thin successive metallic powder layers are applied on a building platform. Each applied powder layer is selectively densified by a high-energy laser beam. The cross-sectional areas (slices) to be densified are calculated from 3D-Models of the desired components. Powder application and densification are repeated until the components are completed [4]. The focused heat input introduced by the laser beam leads to small melt pools, whose heat is quickly dissipated by already solidified material volume. This results in high cooling rates and steep temperature gradients, resulting in high thermal stresses [5]. Acting as a trigger for cracking and component distortion, these stresses can significantly deteriorate component quality and properties [6]. In addition to cracks and component distortion, the quality of PBF-LB/M-manufactured components is influenced by their porosity [7]. Zhang et al. [7] divide the pores formed during PBF-LB/M processing into spherical gas pores and irregular incomplete fusion holes (binding defects). Gas pores are attributed to the outgassing of individual alloy components during melting, which cannot escape due to the fast solidification rates. Binding defects are caused by insufficient melting of the individual melting traces and are mainly found between successive layers or between individual melting traces [7]. The tendency to form defects such as cracks and pores depends on process parameters such as laser power, scanning speed, layer thickness, and on powder properties such as particle size distribution, bulk density, and flowability [7,8]. In addition, the chemical composition of the material to be processed with all its intrinsic properties such as thermal conductivity, heat capacity, latent heat, melting point, viscosity, thermal expansion, gas pressure and absorption, phase transformations, diffusion, and solubility play a significant role in the processability of a material [9].

In the field of steels, alloys that conventionally show good weldability have proved to be processable in PBF-LB/M. These exhibit a sufficiently high deformation capacity to absorb the high thermal stresses caused by microplastic deformation [4]. One of these steels is the austenitic stainless steel X2CrNiMo17-12-2 (AISI 316L, DIN 1.4404). It has a high resistance to corrosion and oxidation, which is why it can serve a wide range of applications, for example, in power plants, in the aerospace industry, or for medical devices such as implants [10,11]. The microstructure (formation) and processability, and the chemical and mechanical properties of steel 316L in the PBF-LB/M condition, were the subject of many studies. Directed growth of elongated grains from the melting line to the center of the melting bath against the maximum temperature gradient was reported [8]. Besides, Yadroitsev et al. [12] detected epitaxial growth in 316L single-track experiments, which is characterized by grains that extend beyond melting lines [12]. Within the grains, a cellular substructure with a high dislocation density can be detected. This is resulting from the rapid solidification and the associated constitutional undercooling [13]. Moreover, a fully austenitic matrix of the PBF-LB/M-manufactured 316L was reported in [8] and [14]. Compared to conventional production routes such as casting, the fine microstructure and the high dislocation density in the PBF-LB/M-processed condition result in an increase in strength [5,15]. 

One aspect that has not yet been considered in the investigations into the PBF-LB/M processing of the 316L steel is the broad tolerance of the alloy limits in accordance with the ASTM A276 standard [16]. As already mentioned, the chemical composition of material influences the PBF-LB/M processing by its intrinsic properties. For this reason, considering the tolerances, there may be different PBF-LB/M processabilities and tendencies for defect formation depending on different chemical compositions for the 316L steel. The type of solidification, for example, depends on the proportion of austenite (C, N, Ni, Mn) or ferrite stabilizing (Cr, Mo, Si) elements and is therefore directly correlated with the chemical composition [17]. It is known from conventional welding of austenitic steels that a primary ferritic solidification increases the resistance against hot cracking. This is due to the fact that the primary solidified ferritic phase binds the elements that promote hot cracking, such as phosphorus and sulfur [18]. The work of David et al. [19] and Vitek et al. [20] had shown that increased cooling rates (higher than conventional welding) lead to changed solidification modalities. One finding was that in 316L steel, the primary austenitic solidification is favored by the fast cooling rates, which results in higher susceptibility to hot cracking [19]. For this reason, it is of significant importance to map the influence of different chemical compositions on the solidification behavior and thus on the tendency to defect formation in the PBF-LB/M processing of steel 316L. In addition, the properties of a material depend on its chemistry, which makes it possible to generate different properties depending on different chemical compositions in the range of permissible alloy limits of 316L, which are shown in Table 1. This work aims to correlate the acceptable alloy limits of steel AISI 316L according to ASTM A276 with the PBF-LB/M processability (tendency to defect formation such as cracks and pores) and the chemical and mechanical properties to finally give a recommendation which alloy composition might lead to an optimum of processability, chemical resistance, and mechanical properties of steel 316L.

It can be concluded that in the PBF-LB/M production of steel 316L, a fine-grained microstructure with a high interfacial density is created due to the rapid solidification and cooling rates. One of the main factors that determine both the solidification in the PBF-LB/M process and the resulting microstructure is the chemical composition of the starting powder.

The research questions to be investigated in this work are:

1. What is the impact of the changes in chemical composition on the processability and the occurrence/types of defects?
2. How is the microstructure formation affected by the variation in the chemical composition (in particular the Cr_eq_/Ni_eq_-ratio) of AISI 316L steel?
3. What is the impact on the corrosion resistance and mechanical properties of the produced parts?
4. Is there a need for a new AM grade standard for type 316L austenitic stainless steel?

## 2. Materials and Methods

### 2.1. Used Starting Materials and Gas Atomization

In this study, three different powder batches of steel AISI 316L are processed. The respective chemical compositions vary within the alloy limits permitted by ASTM 276. The chemical compositions of the three powders used are listed in Table 2. The powder B possesses high Cr and Mo contents while the Ni content is kept low concerning the 316L standard. In contrast, powder C contains a high Ni content and lowered Cr and Mo contents. Powder A shows a middle ground chemical composition between the powder type B and C.

Powder A was purchased from Deutsche Edelstahlwerke GmbH (Witten, Germany). It was produced by closed coupled inert-gas atomization using Ar protection and atomization gas. In contrast, the starting materials B and C were produced by means of inert-gas atomization at the Leibniz-IWT in Bremen, Germany, using a close-coupled atomizer AU 1000 Prototype (Indutherm, Walzbachtal, Germany). Thereby, N_2_ protection gas was used to prevent the melt from oxidation and to atomize it. The usage of N_2_ leads to increased N contents of materials B and C (compare Table 2). The inert-gas atomization of the starting materials was used to achieve spherical particles with median mass diameters in the range of 40–50 µm. The raw materials were melted and superheated to 1715 °C using an indirect inductive melting system. The melt mass-flow for all materials was set to 290 kg/h. For preventing oxidization, the melting chamber was evacuated and subsequently flooded with argon gas. Further details regarding the inert-gas atomization procedure can be found in a previous publication of Ciftci et al. [21].

### 2.2. Powder Feedstock Characterization

The gas-atomized powders B and C were classified by an air classifier type 50 ATP (Hosokawa Alpine, Augsburg, Germany) and an air jet sieve type e200LS (Hosokawa Alpine, Augsburg, Germany)) in the fraction 20–63 μm. Powder A was delivered in the required fraction (20–63 µm) by the manufacturer. The particle size distributions of all powders were analyzed with a diffraction spectrometer type Mastersizer 2000 (Malvern Panalytical Ltd., Malvern, UK). In addition, the particle shape distribution and circularity were measured by dynamic image analysis with iSpect DIA-10 (Shimadzu, Kyoto, Japan). The flowability of the powders was characterized by the Carney method according to ASTM B964-16 (50 g powder, 5 mm nozzle), the Hausner ratio according to ISO 3953, and the angle of repose (prototype machine installed at Leibniz-IWT).

### 2.3. Samples Manufacturing by PBF-LB/M and Sample Designation

All samples were built by means of PBF-LB/M on a cylindrical base plate (type AISI 304 stainless steel) with support structures in an Ar protection gas atmosphere. The used PBF-LB/M device is an AconityMINI (Aconity GmbH, Herzogenrath, Germany). All relevant scanning parameters are given in Table 3.

**Figure 1:**
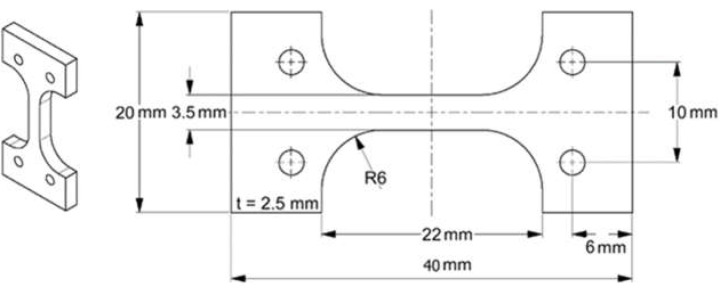
Drawing of a T-bone sample used for the tensile tests.

In the following, powder specimens that were densified by the PBF-LB/M process are designated by the suffix “-PBF”. Stainless steels are often applied in the solution annealed condition to ensure a homogeneous microstructure and high corrosion resistance. In this study, no heat treatment was applied to the investigated specimens since the solution state of the samples manufactured by PBF-LB/M is already high [8]. Thus, all tests were conducted on specimens in the initial as-built state. The specimens used for microstructural investigations and corrosion testing are of a cubic shape with edge lengths of 10 mm. The samples that are used for tensile testing are depicted in Figure 1. Because of their shape, these specimens are referred to as “T-bone” samples. Due to the relatively short shoulder length of the specimens, two holes were designed in each shoulder to fix the specimens during the tensile tests using hardened pins. To avoid premature failure due to stress concentration at the rough specimen surfaces, the surfaces were polished to Rz ≤ 6 µm in accordance with DIN 50125 using SiC abrasive paper and diamond suspension.

### 2.4. Metallography and Microscopy

For microstructural investigations, the cuboid samples manufactured by PBF-LB/M were embedded in an electrically conductive resin. Subsequently, the top and bottom surfaces of the samples were stepwise ground with SiC abrasive paper from 320 to 1000 mesh and finally polished with diamond suspensions with grain sizes from 3 to 1 μm. For increased visibility of the microstructures, the metallographically prepared cross-sections were etched with V2A etching solution (100 cm^3^ H_2_O, 100 cm^3^ HCl, 10 cm^3^ HNO_3_, 1 cm^3^ Vogel’s special reagent). 

The relative density of the PBF-LB/M samples was determined by image analysis using an optical microscope type BX60M (Olympus, Tokyo, Japan) and the software ImageJ (v1.52p) (National Institute of Health, Bethesda, MD, USA). For calculating the relative density, the accumulated area of all pores in the measuring area was set in relation to the extraction area. Each of the investigated samples was prepared in three different layers. The density of the sample was determined from the mean value of the densities of these three layers. 

To investigate the particle morphology of the powders (non-polished particles attached to adhesive carbon tape), as well as the microstructures of the PBF-LB/M-processed samples, a field emission scanning electron microscopy (SEM) of type MIRA3 (TESCAN, Brno, Czech Republic) was used. The SEM was operated at an acceleration voltage of UA = 15 kV at a working distance of WD = 15 mm.

### 2.5. Thermodynamic Calculations

For thermodynamic calculations, the ThermoCalc software (ThermoCalc AB, Stockholm, Sweden) in version 2021a was used. Calculations were performed with the database TCFE10 while following phases were selected: LIQUID (liquid), FCC_A1 (γ-Fe), BCC_A2 (δ-Fe, α-Fe), M23C6 (Cr-rich M_23_C_6_), M7C3 (Cr-rich M_7_C_3_), HCP_A3#2 (Cr-rich M_2_N), SIGMA (Cr-rich ς-Phase) and Laves_Phase (Laves phase C14). All calculations were performed at atmospheric pressure of 1000 mbar and a substantial quantity of 1 mol. To illustrate the solidification sequence of the steel, solidification simulations according to the approach by Scheil and Gulliver with the thermodynamic database TCFE10 were performed. These calculations were also carried out at atmospheric pressure of p = 1000 mbar and a quantity of 1 mol, starting from a temperature of 2500 °C. The temperature was reduced by 1 K steps. Fast diffusion of the elements C and N was taken into account.

### 2.6. Phase Analysis by X-ray Diffraction Experiments and Magneto Inductive Measurements

To identify the phases formed in the PBF-LB/M-manufactured specimens, X-ray diffraction (XRD) measurements were carried out on metallographically prepared surfaces of the specimens using a Bruker D8 Advanced device (Bruker, Billerica, MA, USA) with a Bragg–Brentano setup. CuK_α_ radiation with a wavelength of 1.5406 Å was used for the measurements in a range of 30–90° 2θ with a step size of 0.01° and an acquisition time of 2 s per increment. The specimens were rotated during the measurement. The XRD patterns were analyzed using the DIFFRAC.EVA V3.0 software (Bruker, Billerica, MA, USA) with the JCDPS PDF-2 database.

In addition, the ferrite content of the specimens was determined using Rietveld analysis [22]. Therefore, the analysis software MAUD was used to process the XRD patterns gathered from the Bruker D8 Advanced XRD device [23,24]. The phase evaluated was bcc ferrite (COD-file 9008536). Reference measurements were carried out using an Al_2_O_3_ reference material (COD-file 1000032). 

Besides the Rietveld method, the ferrite content was evaluated by using a magneto-inductive Feritscope FMP30 device (Helmut Fischer GmbH, Sindelfingen, Germany) according to DIN EN ISO 17655. Thereby, the content of the ferromagnetic bcc ferrite phase is determined by measuring the magnetic permeability of a half-sphere volume with a radius of 2 mm [25]. The measuring probe was applied five times to both the top and the bottom surfaces related to the building direction of the additively manufactured samples. The mean value of the five measurements was calculated. The FMP30 device was calibrated for measuring the ferrite content in a range of 0.1 to 80 vol.% using standard samples according to DIN EN ISO 8249. 

### 2.7. Corrosion Testing

The uniform surface corrosion resistance of the PBF-LB/M-manufactured samples was tested in 0.5 mol (5%) sulfuric acid (H_2_SO_4_) using a corrosion testing cell in accordance with ASTM G5. The samples were tested in the as-built condition. An austenitic steel wire was spot-welded to the bottom of the cubic PBF-LB/M-produced samples before embedding in non-conducting polymer resin. The embedded samples were stepwise ground with SiC abrasive paper up to 1000 mesh. To prevent crevice corrosion between the sample and the embedding resin and to avoid unintended electrical contact, the sample and the wire were covered with a varnish, leaving only a square surface of the steel specimen exposed. This exposed area was measured using a flatbed scanner to calculate the current density inside the corrosion testing software (Ivium, Eindhoven, The Netherlands). In the corrosion testing setup, the sample is treated as a working electrode connected to a Pt sheet counter electrode and a saturated calomel electrode (SCE, +244 mV vs. standard hydrogen electrode) as reference. The potentials measured during this study are given relative to a calomel reference electrode. All tests were performed using a Vertex potentiostat (Ivium, Eindhoven, The Netherlands) at room temperature. Before corrosion testing, the used electrolyte was purged with nitrogen gas for 30 min to ensure a comparable amount of oxygen at the beginning of each test. Each specimen was then cathodically polarized at −2 V for 60 s to remove the native oxide layer from the specimen surface. The open-circuit potential (OCP) was measured for 30 min, during which time the oxide layer subsequently reformed due to the oxygen remaining in the electrolyte. With respect to the oxygen content of the starting powders and the built specimens, a similar number of non-metallic inclusions which can act as local elements is present. Thus, comparability between the respective specimens A-PBF, B-PBF, and C-PBF is guaranteed. The polarization curves were recorded with an initial potential of 10 mV under the previously measured OCP and a potential step rate of 0.167 mV/s. Each measurement was performed twice on different cubic samples.

### 2.8. Mechanical Testing

To investigate the mechanical properties of the three PBF-LB/M-processed materials specimens, tensile tests were performed for each material at room temperature according to DIN 50106. For each PBF-LB/M-densified material A, B, and C, five individual tensile specimens were tested. The tensile tests were performed using a servohydraulic Instron PSB100 testing system (Instron, Norwood, MA, USA) with a 75 kN load cell attached to the tensile specimens (see Figure 1) with the applied load being perpendicular to the building direction of the PBF-LB/M process. The strain measurement was carried out using a tactile extensometer (Instron, Norwood, MA, USA) with a gauge length of 10 mm and a strain measurement range of ±10%.

Besides, Vickers hardness testing was performed at the ground and polished cuboid specimens in accordance with DIN EN ISO 6507-1 using a KB30s hardness testing device (KB Prüftechnik GmbH, Hochdorf-Assenheim, Germany). The load was set to 9.807 N (HV1), and the arithmetical mean of five measurements was calculated.

## 3. Results

### 3.1. Powder Characteristics

**Figure 2:**
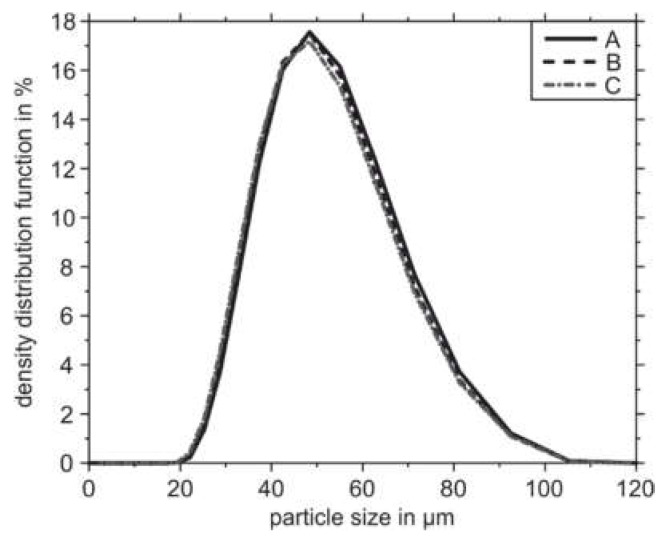
Particle size density distribution functions.

To ensure the comparability of the used powder batches A, B, and C, the characteristic powder properties of each powder were evaluated. Figure 2 shows the particle size density distributions of the three starting powders A, B and C. All three powders do not show major differences concerning the particle size distribution measured using the diffraction spectrometer. The values d(0.1), d(0.5), and d(0.9) are set around 31 µm, 45 µm, and 64 µm for the used powders A, B, C, respectively. 

In addition, the characteristic powder properties are given in Table 4. The flowability characterized by the Carney flow time, the angle of repose, and the Hausner ratio does not show significant differences regarding powders A and C. However, starting powder B shows inferior flowability than powders A and C concerning the Carney flow time and the achieved angle of repose. However, the Hausner ratio of 1.18 is similar to the values of the starting powders A and C. Consequently, a dense powder layer application could be achieved for all the starting powders A, B, and C. 

**Figure 3:**
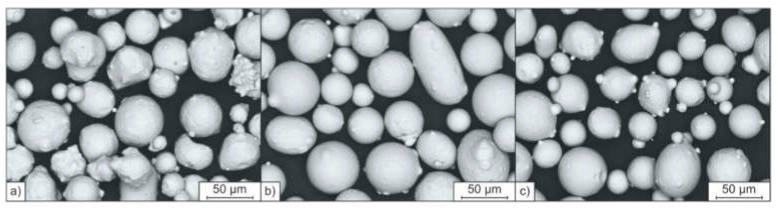
Morphologies of the starting powders; (**a**) A, (**b**) B, (**c**) C.

Besides, Figure 3 shows that the used starting powders also possess a similar morphology compared to each other. Among high spherical particles, few elongated particles can be found. Moreover, powder particles containing satellites can be observed within the starting powder feedstocks. 

**Figure 4:**
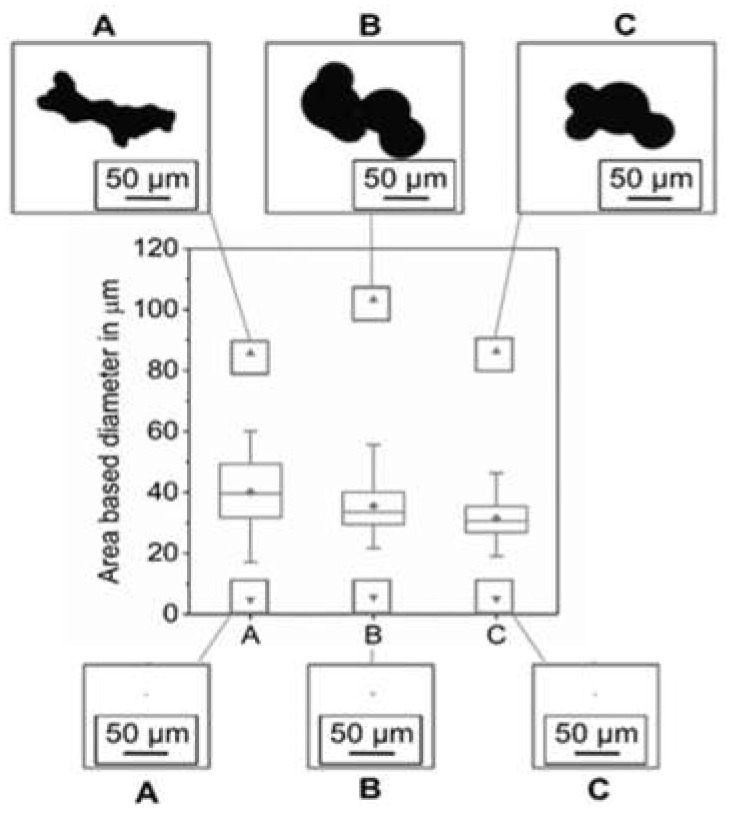
Particle shape distributions.

Regarding the particle shape distribution measurement using dynamic image analysis, powder A shows a higher range in particle size and scatter than the other two starting materials (Figure 4). The minimum powder sizes for the three powders are given in Table 4. The maximum particle size was measured for powder B. For all three starting materials, the largest powder particles result from the agglomeration of the smaller particles during the atomization process (satellites). However, in the case of powder A, the agglomerated powder particles possess sharper corners, suggesting that they resulted from the agglomeration of smaller powder particles compared to the B and C powders. Powders B and C show relatively bulkier agglomerated particles owing to the different gas atomization setup that was used in comparison to powder A. The largest agglomerates are found for powder B. This can be a reason for the increased Carney flow time and angle of repose of powder B. It can be assumed that the different chemical compositions of powders B and C influence the atomization process.

### 3.2. PBF-LB/M Densification and Microstructures

**Figure 5:**
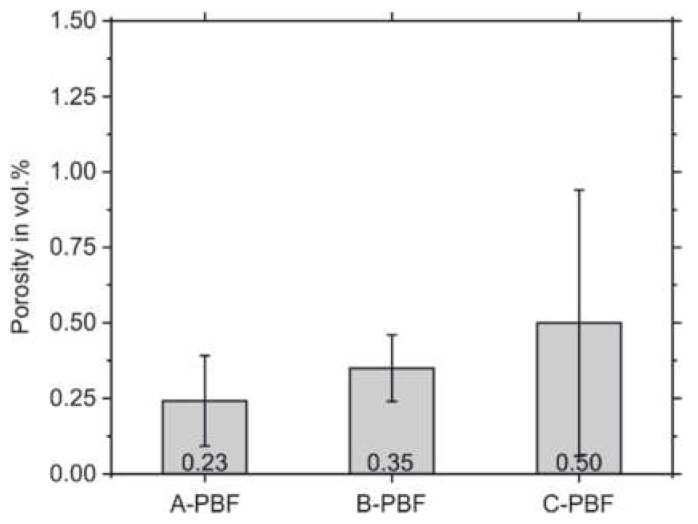
Porosities of the PBF-LB/M-processed samples.

After PBF-LB/M-processing of the three starting powders, the remaining porosity of the cuboid specimens was determined by quantitative image analysis. The results are presented in Figure 5. By applying the standard PBF-LB/M parameter set recommended by the system manufacturer (Table 3), the samples, A-PBF, show the lowest porosity of 0.24% compared to the samples B-PBF and C-PBF. The samples B-PBF and C-PBF, which possess chemical compositions that are set at the boundaries of the allowed Cr and Ni equivalents (Cr_eq_ and Ni_eq_), respectively, show higher porosities of 0.35% and 0.50%. The densities after PBF-LB/M cannot solely be related to the flow properties of the powders that are presented above. The evaluated powder properties seem to be less influencing the microstructure formation during the PBF-LB/M densification process. Thus, the different chemical compositions preferably determine the microstructure formation.

**Figure 6:**
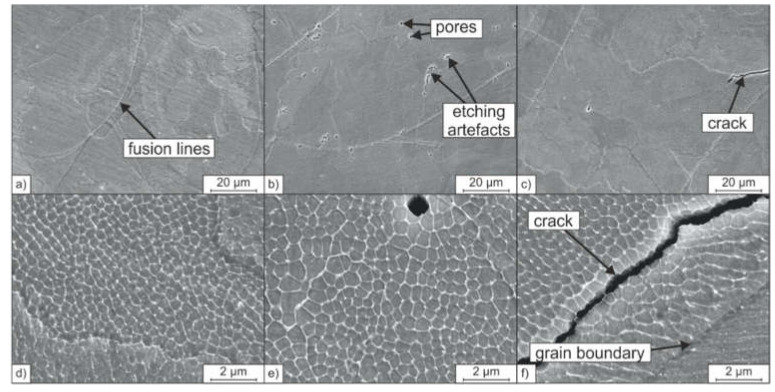
SEM micrographs of the microstructures of the samples: (**a**) A-PBF, (**b**) B-PBF, (**c**) C-PBF at low magnification; (**d**) A-PBF, (**e**) B-PBF, (**f**) C-PBF at high magnification.

Figure 6 shows the associated microstructures of the PBF-LB/M-densified samples. All samples, A-PBF, B-PBF, and C-PBF, show a hierarchical microstructure as it was also reported for PBF-LB/-manufactured 316L steel in previous studies [8,26,27]. Fusion lines resembling the laser-generated melt pools and large-angle grain boundaries can be observed on the mesoscale. The grains grow epitaxially in the direction of the heat gradient during the layer-wise built-up. Thereby, the crystallographic orientation of the mother grains is passed on to newly solidifying grains at the fusion lines [27]. At higher magnifications, a fine cellular substructure inside these grains or colonies can be observed (see Figure 6d–f). Krakhmalev et al. [27,28] showed that the cells inside the respective colonies share a continuous crystallographic plane. Therefore, the borders between adjacent cells should not be interpreted as additional large-angle grain boundaries. Still, the borders between the cells are characterized by a high dislocation density, as transmission electron microscopy investigations made by Voisin et al. and others showed [8,27,29]. The formation of the cellular substructure is based on the rapid solidification rate resulting from the typical small melt pools generated during the PBF-LB/M process. This is also associated with high thermal gradients and thermal stresses [8,27]. In addition, the areas between the cells are prone to microsegregation of the alloying elements such as Cr and Mo [8,27].

Although the obtained microstructures of the samples A-PBF, B-PBF, and C-PBF appear to be similar, differences can be found in the formation of volumetric defects. The defects found in all the samples are primarily spherical pores and few irregularly shaped binding defects. Binding defects can be traced back to voids in the applicated powder bed and insufficient local wetting owing to insufficient energy input [7,30]. Spherical pores can be related to evaporation or outgassing of chemical elements due to excessive local energy inputs [31]. The element N can recombine to gaseous N_2_ and thus forms spherical gas pores during PBF-LB/M [32]. This behavior can be one reason for the higher porosities of B-PBF and C-PBF compared to the material A-PBF. Powders B and C both possess higher N contents than material A (compare Table 2). 

Furthermore, unlike the samples A-PBF and B-PBF, the sample C-PBF shows cracks that reduce the relative density (Figure 6). The cracks run intergranular through the cellular substructure, as can be seen in Figure 6f. Cracks in austenitic steels are often associated with hot cracks [33]. However, the cracks found cannot be interpreted as hot cracks, as these are formed on the large-angle grain boundaries owing to low melting phases (solidification cracks) [34]. The intergranular crack morphology found in sample C-PBF is a result of solid-state cracking. Moreover, the contents of the hot crack promoting elements S and P, which form low melting phases, are low in starting powder C (0.01 mass% each, compare Table 2).

Solid-state cracking in austenitic steels is reported in welding technology [35]. A solid-state crack type known from austenitic and Ni-base welds is the so-called ductility dip crack (DDC). Theories for the DDC formation are provided by Lippold et al. [36]. DDCs occur in a temperature range between 60% and 90% of the solidus temperature (T_SOL_; approximately 800° to 1150 °C in austenitic steels). The crack mechanism is a namesake dip in the material’s ductility in this temperature range. The ductility is reduced due to grain growth and sliding below the recrystallization temperature, leading to the localization of tensile stresses at the grain boundaries [37,38,39]. If these stresses exceed the material’s strength at the grain boundaries, crack formation at the grain boundaries takes place. Owing to multiple heating during subsequent weld passes in multilayer welds or additive manufacturing processes such as PBF-LB/M, the crystallographic grain boundaries move away from the initial solidification-related grain boundaries. For this reason, DDCs typically occur in a mostly straight line next to the original grain boundary at a migrated crystallographic grain boundary. This behavior can also be observed in Figure 6f for the sample C-PBF. The crack runs parallel to the solidification-related grain boundary at a distance of approximately 3 µm. 

It is assumed that the cracks formed in sample C-PBF are DDC. The other samples, A-PBF and B-PBF, do not show cracks while the starting powder properties are comparable, and the PBF-LB/M process parameters stayed untouched. Therefore, the reason for the DDC formation must be ground in the specific chemical composition of powder C as this is the only significant difference between the used starting powders. Powder C possesses a higher Ni_eq_ compared to powders A and B (Table 2). Therefore, the fcc austenite is stabilized at the cost of the stability of the bcc ferrite phase [40]. 

Alloys that consist of only one phase (e. g., only austenite) in the temperature range of the ductility dip and do not form precipitates at the grain boundaries are highly susceptible for DDC. Such alloys suffer from increased grain growth and sliding, promoting stress localization at the grain boundaries [37,38,39]. 

**Figure 7:**
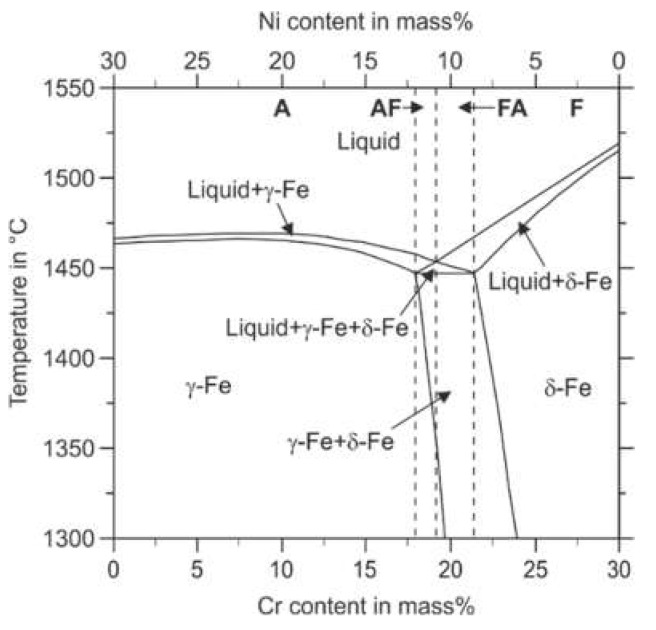
Pseudobinary section of the phase diagram of the alloying system Fe–Cr–Ni at 70 mass% Fe calculated using ThermoCalc.

Additively manufactured 316L stainless steel is known for being fully austenitic in the PBF-LB/M processed condition [41]. However, the solidification path and phase formation in the solid-state of 316L steel can also include the bcc phase ferrite [42]. Figure 7 illustrates the thermodynamically stable phases in the Fe–Cr–Ni system concerning the Cr and the Ni content, respectively. Fe–Cr–Ni alloys can solidify in four different modes called A, AF, FA, and F. Mode A means primary austenitic solidification with no ferrite involved. According to the thermodynamic calculation, this mode occurs for lower Cr to Ni ratios. According to Faulkner et al., in welding processes, the fully austenitic solidification mode A is enabled for steels that possess a Cr_eq_/Ni_eq_ ratio smaller than 1.3 [43]. Higher Cr_eq_/Ni_eq_ ratios result in primary austenitic solidification with crystallization of ferrite in the latter solidification path (AF-Mode). However, the ferrite stability decreases with decreasing temperature leading to a ferrite to austenite transformation in the solid-state during the cooling period. The intrinsic heat treatment during the deposition of subsequent material layers in the PBF-LB/M process can promote such phase transformations during the build-up of a component [44]. The further increased Cr_eq_/Ni_eq_ ratios enable the FA solidification mode. In the FA-mode, ferrite crystallizes primarily from the melt, followed by the austenite phase. However, due to decreasing stability of the phase ferrite with decreasing temperature, the FA-Mode can also result in a fully austenitic microstructure at room temperature (compare Figure 7). A further increased Cr_eq_/Ni_eq_ ratio leads to a fully ferritic solidification when the mode F is entered. 

**Figure 8:**
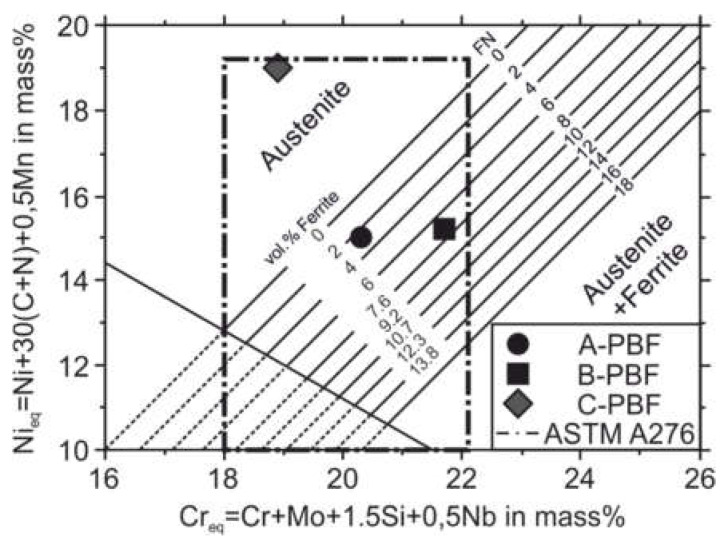
Representation of the DeLong diagram according to [1] with positions of the used starting materials.

To estimate the obtained austenite and ferrite contents of the solidified material concerning the Cr_eq_ and Ni_eq_ after welding processes (fast non-equilibrium solidification), the diagrams of Schaeffler and DeLong were introduced [40,45]. Figure 8 shows the positions in the DeLong diagram of the three investigated sample alloys A, B, and C within the compositional range of the AISI 316L steel. According to the DeLong diagram, the wide compositional range allowed for steel 316L by the ASTM A276 standard can promote fully austenitic and austenitic-ferritic microstructures. A fully austenitic microstructure is predicted for alloy C, while for the alloys A and B ferrite contents of approximately 2.5 vol.% and 6.5 vol.%, respectively, are predicted.

**Figure 9:**
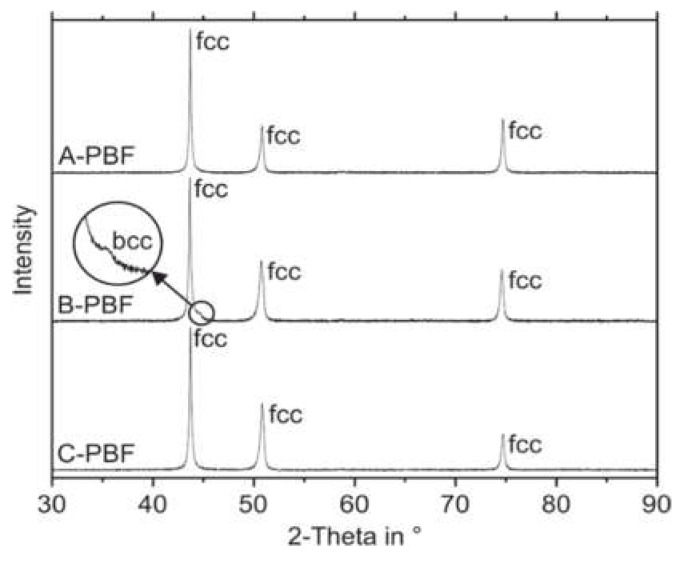
XRD patterns of the PBF-LB/M samples.

In contrast to the predictions according to the DeLong diagram, XRD measurements reveal a fully austenitic microstructure not only for the sample C-PBF but also for the sample A-PBF (Figure 9). The XRD pattern of the sample B-PBF shows a small reflection of the bcc phase. 

The patterns shown in Figure 9 were measured at the ground top surface of the specimens. XRD measurements were also performed at the ground bottom surfaces of the samples to investigate a possible effect of the intrinsic heat treatment during the PBF-LB/M process on the ferrite contents. As these patterns appear similar to those obtained from the top surfaces, they are neglected in Figure 9 for reasons of clarity. The actual ferrite contents of the samples were calculated from the XRD measurements by the Rietveld method for both the top and bottom surfaces. The results are given in Table 5.

Additionally, magneto-inductive measurements of the ferrite contents were conducted using the Feritscope. The magneto-inductive measurements confirm the fully austenitic microstructure for the samples A-PBF and C-PBF. Furthermore, the Feritscope detects ferrite in the sample B-PBF, which is also in accordance with the results of the Rietveld analysis. The Rietveld analysis and the magneto-inductive measurements show increased ferrite contents at the top surface (0.5, respectively, 0.72 vol.%) compared to the bottom surface (0.18, respectively, 0.38 vol.%). These local differences in the ferrite content indicate that the intrinsic heat treatment during the repeated deposition of material layers promotes a transformation of primarily solidified ferrite to austenite in the underlying layers of the sample B-PBF.

The assumed transformation of the primarily solidified ferrite into austenite in the sample B-PBF that possesses a high Cr_eq_/Ni_eq_ ratio during the further heat input also stands in accordance with an observation made in previous works regarding the rapid solidification of austenitic steels, such as in the PBF-LB/M process [19,20]. David et al. [19] reported a shift from the mixed solidification modes (AF and FA) to the fully austenitic, respectively, fully ferritic solidification mode for higher solidification rates. High solidification rates counteract the compositional balancing by diffusion processes during solidification because these diffusion processes are dependent not only on temperature but also on time (kinetic). In general, the phase that crystallizes first is stabilized by rapid solidification. With the deposition of subsequent layers during the component build-up in PBF-LB/M, the rapid and almost diffusionless solidified material is heated. This heat can then enable diffusion processes and promote phase transformations so that more stable phases at lower temperatures are formed [44,46,47]. This effect increases for earlier deposited layers as these are affected by more depositions of subsequent layers.

**Figure 10:**
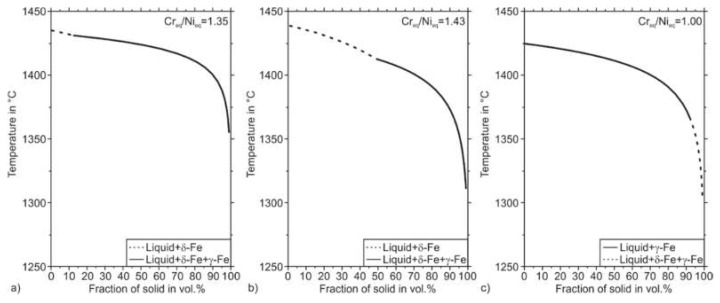
Solidification paths calculated using the Scheil–Gulliver approach; (**a**) A-PBF, (**b**) B-PBF, (**c**) C-PBF.

As shown so far, the rapid solidification in PBF-LB/M processing cannot be depicted by the DeLong diagram. However, a non-equilibrium and diffusionless solidification similar to the conditions during PBF-LB/M can be simulated by thermodynamic calculations after the approach by Scheil and Gulliver. The diffusionless solidification paths of the samples A-PBF, B-PBF, and C-PBF, calculated after the Scheil-Gulliver approach, are depicted in Figure 10. The solidification simulations predict a primary solidification of bcc ferrite for the samples A-PBF and B-PBF, which possess Cr_eq_/Ni_eq_ ratios of 1.35 and 1.43. For sample C-PBF containing a Cr_eq_/Ni_eq_ ratio of 1.00, primary solidification of fcc austenite is predicted. The solidification simulations are in accordance with the results of Faulkner et al., who stated fully austenitic solidification of steels for Cr_eq_/Ni_eq_ ratios smaller 1.3 [43]. However, the microstructure of the PBF-LB/M solidified sample A-PBF is fully austenitic and does not include the bcc ferrite phase (compare Table 5). The aforementioned intrinsic heat treatment can promote the absence of the phase ferrite in the sample A-PBF during the PBF-LB/M process, which allows the solidified ferrite to transform into austenite. However, as the top layers that were exposed to less or no intrinsic heat treatment are also free from the phase ferrite, it could be assumed that contrary to the Scheil–Gulliver simulation, the sample A-PBF already solidified fully austenitic. Kelly et al. also found a change from the FA solidification mode to the fully austenitic mode A instead of the mode F for rapid solidification and high undercooling of the melt [48]. They related this behavior to a preferred crystallization of the fcc austenite owing to its formation kinetics.

It can be concluded that the microstructure formation of austenitic steels during PBF-LB/M is complex and, therefore, can hardly be predicted by the empirical approach of DeLong or by Scheil–Gulliver’s simulations. The alloys A and C with Cr_eq_/Ni_eq_ ratios of 1.35, respectively, 1.00, showed a fully austenitic microstructure after PBF-LB/M densification. While the alloy B with a higher Cr_eq_/Ni_eq_ ratio of 1.43 showed small amounts of ferrite (0.18–0.72 vol.%). In contrast to the fully austenitic sample A-PBF, the fully austenitic sample C-PBF with the lowest Cr_eq_/Ni_eq_ ratio of 1.00 showed DDC in the as-built state. It is evident that Cr_eq_/Ni_eq_ ratios as low as 1.00 should be avoided even if the susceptibility for solidification cracking (hot cracking) is eliminated by low P and S contents to prevent the crack formation at all (compare Table 2).

### 3.3. Corrosion Resistance of the PBF-LB/M Densified Powders

**Figure 11:**
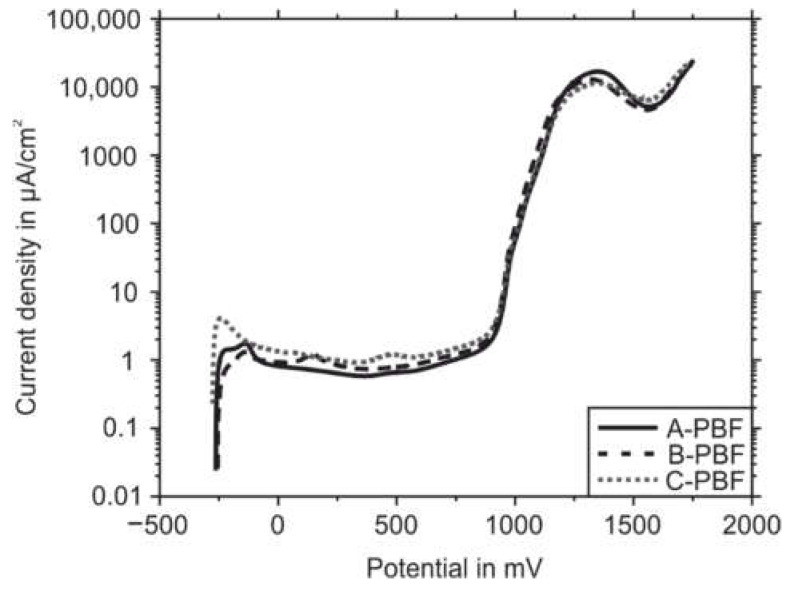
Current density/potential curves of the PBF-LB/M-processed samples.

In Section 3.2, a connection between the microstructure formation and the microstructural defects depending on the different chemical compositions of the steel AISI 316L starting powders were pointed out. Now, these findings are to be linked with the corrosion behavior of the different variants represented in Figure 11 and Table 6, respectively. These show the current density/potential curves and the associated characteristic values of the materials under investigation. It can be stated that the results do not differ significantly concerning the detected characteristic potentials. Only slight differences can be seen in the passivation and passive current density. These values correlate with the anodic metal dissolution during the corrosion attack and therefore represent a direct indicator of the corrosion resistance of the respective material. The lowest metal dissolution and the highest corrosion resistance can be seen in the sample A-PBF, followed by B-PBF and C-PBF. The difference in the corrosion behavior of the three 316L variants could be attributed to the microstructural defects described in Section 3.2. The higher the density of microstructural defects such as cracks and pores, the greater the specific surface area exposed to the corrosive attack. Consequently, higher passivation and passive current densities can be found for materials with lower relative density, such as the samples B-PBF and C-PBF in this study [49,50]. In addition, Otereo et al. [51] showed that pitting and crevice corrosion phenomena can occur within pores, which can also have a negative impact on resistance to uniform surface corrosion. This is particularly favored by a deficient electrolyte exchange within the pore, which leads to the depletion of the oxygen necessary to maintain the passive layer. This effect could be even more pronounced in cracks such as those found in sample C-PBF because of the higher aspect ratio of a crack than a spherical pore, making electrolyte exchange even more difficult. 

In addition to the defect density, the chemical composition is also a significant factor influencing the corrosion resistance of stainless steel. For instance, Yu et al. [52] found that an increased Cr content leads to lower passivation and passive current densities due to the formation of a more stable passive layer. Table 2 shows that the material C-PBF has the lowest Cr content of the steels examined. Accordingly, in addition to the higher defect density, the inferior corrosion resistance of C-PBF could be attributed to the lower Cr content. 

It can be assumed that the effects of the higher defect density and the lower Cr content of the sample C-PBF and the associated worse corrosion behavior are superimposed. Therefore, it is not possible to specify which influence is responsible for the lower corrosion resistance of C-PBF exactly. However, it can be stated that the chemical composition of the steel processed by PBF-LB/M is directly related to the chemical properties. This is based on the one hand on chemical composition-dependent formation and stability of the passive layer, and on the other hand on the susceptibility, dictated by the chemical composition, to form microstructural defects such as solid-state or solidification cracks. 

### 3.4. Mechanical Properties of the PBF-LB/M Densified Powders

The chemical composition and the microstructure are related to the composition and the processing route of a material and affect, not only the corrosion resistance but also the mechanical properties of components. To evaluate the mechanical properties of the three investigated 316L variants A, B, and C in the PBF-LB/M-processed condition, tensile and hardness testing were performed. Because the starting powder properties were comparable and the PBF-LB/M process parameters were kept constant for all samples produced, the differences in the mechanical behavior can be entirely related to the changes in the chemical composition and the associated changes in the microstructure formation.

**Figure 12:**
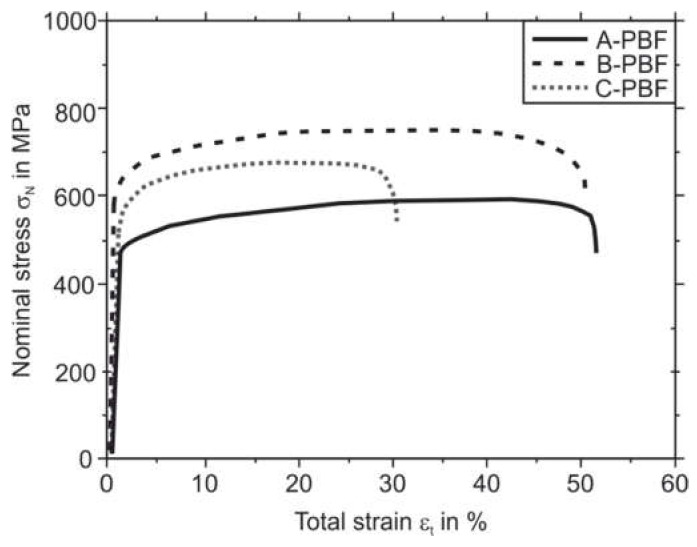
Stress/strain curves of the PBF-LB/M-processed T-bone samples.

Figure 12 shows the results of the quasi-static tensile tests of the PBF-LB/M specimens. For every sample type, A-PBF, B-PBF, and C-PBF, the averaged curves of three tested samples are plotted. Sample B-PBF shows the highest strength with a yield strength R_p0.2_ of 593 MPa and ultimate tensile strength R_m_ of 7522 MPa. The sample A-PBF possess the lowest yield strength R_p0.2_ and tensile strength R_m_ of 465 MPa, respectively, 564. MPa. The samples A-PBF and B-PBF show almost identical elongations at fracture A_5_ of 5.1% and 5.1%, respectively. Specimen A-PBF shows the lowest A_5_ of 3.0%. The characteristic values obtained from the tensile test and the hardness tests are collected in Table 7. The hardness of the PBF-LB/M specimens corresponds to the strength of the samples. Consequently, the sample B-PBF shows the relatively highest hardness of approximately 253 HV1, followed by the samples C-PBF (234 HV1) and A-PBF (184 HV1).

Considering the chemical compositions of the samples, which is given in Table 2, the increased hardness and strength of the samples B-PBF and C-PBF compared to A-PBF can be ground in the increased contents of the elements C and N. These elements can be dissolved interstitially in the metal matrix and can therefore cause a strong solid-solution hardening effect [53]. The strengthening effect of increased N contents in additively manufactured 316L stainless steel was also observed in a previous study by Boes et al. [32]. The formation of nitrides was suppressed by the rapid solidification rate present in the PBF-LB/M process so that the increased N contents were dissolved only in the metal matrix [32]. Boes et al. investigated nitrogen contents of 0.1 and 0.3 mass% and found a slight decrease in the elongation at fracture from 47.5% in a low nitrogen (0.03 mass%) 316L reference to 41.7% and 36.4%, respectively [8,32]. They attributed the loss in elongation at fracture in case of the high N content to an increase in porosity associated with the increased N contents. High N contents will promote the formation of gas pores due to the outgassing of recombined N_2_ if the solubility in the laser-generated melt pool is exhausted. The N solubility decreases with increasing overheating of the melt pool [54]. 

A decrease in the elongation at fracture can also be observed for C-PBF. The reason for this can also be given by an increased porosity (compare Figure 5). However, this increase in porosity is not mainly linked to the outgassing of N_2_ but also to the formation of solid-state cracks, namely the DDCs. However, it has to be mentioned that the increased Ni content of the starting material C can promote the outgassing of N_2_ owing to a reduction in N solubility associated with increased Ni contents [32,54]. The main reason for the reduction in elongation at fracture for C-PBF is assumed to be the occurrence of the DDCs. These cracks provide a more pronounced notch effect than roundly shaped pores. Although increased contents of N and C increase the strength of the additively manufactured 316L steel by solid-solution hardening, these alloying elements also lead to an increase in the Ni_eq_. According to the DeLong diagram, the elements N and C increase the Ni_eq_ 30 times stronger than Ni (see Figure 8) [40]. The strong increase in the Ni_eq_ then promotes the formation of the DDCs, which negatively affects the mechanical properties.

### 3.5. Derivation of an Adapted Compositional Range of the Austenitic Stainless Steel Suitable for PBF-LB/M Processing

To avoid solid-state cracking and to utilize the strengthening effect of increased N contents simultaneously, it is suggested to restrict the Ni_eq_ by lowering the maximum Ni content. This can also prevent the pronounced appearance of gas pores by N_2_ outgassing. The Cr_eq_/Ni_eq_ ratio of a 316L type stainless steel in PBF-LB/M production should be higher than 1.3 to prevent the formation of DDCs, which otherwise can negatively affect both the corrosion resistance and the elongation at fracture of the additively manufactured components.

Based on the ASTM A276 standard, a possible adaption of the compositional range of 316L type stainless steel is given in Table 8. To limit the Cr_eq_/Ni_eq_ ratio to values higher 1.3 by lowering the Ni content, a maximum Ni content of approximately 10 mass% is considered.

In addition, the recommended range of the Cr-content is narrowed to a range of 17 to 18 mass%. Consequently, the compositional range given in Table 8 leads to a minimum Cr_eq_/Ni_eq_ ratio of 1.28 and a maximum Cr_eq_/Ni_eq_ ratio of 2.21. In comparison, the minimum Cr_eq_/Ni_eq_ ratio that can result from the compositional range given by the ASTM A276 is 1.01. The maximum Cr_eq_/Ni_eq_ ratio is also 2.21. It is to mention that Papula et al. and Hengsbach et al. reported ferritic microstructures for PBF-LB/M-processed stainless steels with Cr_eq_/Ni_eq_ ratios higher than 2.25 [55,56]. Therefore, the upper limit of the Cr_eq_/Ni_eq_ ratio must be evaluated to give a more precise recommendation on a suitable compositional range of 316L type stainless steel adapted for PBF-LB/M processing. Additionally, the influence of the PBF-LB/M process parameters on the solidification rates and solidification paths with respect to the changes in the solidification mode of Cr–Ni steels must be investigated more deeply in future works. 

## 4. Conclusions

In this study, three variants of the stainless steel AISI 316L that differ in their Cr_eq_/Ni_eq_ ratios were processed by means of PBF-LB/M. After PBF-LB/M processing, the samples were characterized regarding their microstructure and defect formation. Furthermore, the corrosion resistance and the quasi-static mechanical properties were evaluated. The following statements can be drawn from the experimental results:

1. The starting material with the highest Ni_eq_ owing to increased contents of the alloying elements Ni and N resulted in the highest porosity after the PBF-LB/M processing. The reduction of the Cr_eq_/Ni_eq_ ratio to 1.00 promoted the formation of solid-state cracks. These cracks are identified as ductility dip cracks. Their formation is ground in the fully austenitic solidification path;
2. The solidification path seems to change between austenitic–ferritic and fully austenitic solidification mode in PBF-LB/M processing if the Cr_eq_/Ni_eq_ ratios are changed within the allowed range given by the ASTM A276 standard for the steel 316L. The obtained microstructures cannot be predicted by the DeLong diagram. The alloy with the highest Cr_eq_/Ni_eq_ ratio of 1.43 showed small amounts <1 vol.% of ferrite in the as-built state. The ferrite content is reduced by the intrinsic heat treatment at the bottom parts of the sample;
3. The formation of defects caused by the low Cr_eq_/Ni_eq_ ratio of 1.00 decreases the corrosion resistance due to an increase in surface area and promotion of crevice corrosion phenomena;
4. Furthermore, the formation of cracks promoted by the low Cr_eq_/Ni_eq_ ratio of 1.00 decreases the elongation at fracture;
5. In PBF-LB/M processing, Cr_eq_/Ni_eq_ ratios should be restricted to values higher than 1.30. Thereby, the Ni content should be limited in particular to ensure the crack-free PBF-LB/M production of 316L type steel. Reduced Ni contents enable the usage of higher N contents without promoting the formation of gas pores by the outgassing of N_2_. A recommendation on more suited alloy limits is given.

## Figures and Tables

**Table 1:** Alloy range in mass% permitted according to ASTM A276 for the steel AISI 316L [16].

	C	Si	Mn	P	S	Cr	Mo	Ni	N
min.	-	-	-	-	-	16.0	2.00	10.0	0.00
max.	0.03	0.75	2.00	0.045	0.03	18.0	3.00	14.0	0.10

**Table 2:** Middle chemical compositions of the used materials in mass%.

Material	C	Si	Mn	Cr	Ni	Mo	N	P	S	O	Cr_eq_	Ni_eq_	Cr/Ni
A	0.01	0.83	0.72	16.55	13.38	2.46	0.03	0.02	0.02	0.03	20.27	15.04	1.35
B	0.03	0.79	1.71	17.46	10.13	3.01	0.11	0.01	0.01	0.02	21.66	15.18	1.43
C	0.03	0.82	1.73	15.53	13.88	2.17	0.11	0.01	0.01	0.02	18.99	18.93	1.00

**Table 3:** Used PBF-LB/M process parameters.

Laser Power in W	Scanning Speed in mm/s	Layer Thickness in µm	Hatch Distance in µm	Laser Spot Size in µm	Scan Strategy	Tilt Angle	Protection Gas
150	800	30	80	50	stripes (10 mm)	37°	Ar

**Table 4:** Characteristic powder properties of the used starting powders.

Powder	Carney Flow Time in s	Angle of Repose in °	Hausner Ratio	Minimum Particle Size in µm	Maximum Particle Size in µm
A	3.5	32	1.22	4.77	85.64
B	8.5	54	1.18	5.59	103.26
C	4.0	40	1.20	5.06	86.26

**Table 5:** Ferrite contents of the PBF-LB/M-processed samples.

Sample	Position	Ferrite Content in vol.%	
		Magnetic Measurement	Rietveld Method
A-PBF	top	0.00	0.00
	bottom	0.00	0.00
B-PBF	top	0.72 ± 0.07	0.50 ± 0.07
	bottom	0.38 ± 0.02	0.18 ± 0.00
C-PBF	top	0.00	0.00
	bottom	0.00	0.00

**Table 6:** Results of current/potential tests performed on the three PBF-LB/M-processed 316L variants in the as-built condition.

Material	Open Circuit Potential in mV	Passivation Potential in mV	Passivation Current Density in µA/cm^2^	Activation Potential in mV	Breakdown Potential in mV	Passive Current Density in µA/cm^2^
A-PBF	−262	−214	1.74	−62	918	0.76
B-PBF	−255	−142	1.30	−68	908	0.91
C-PBF	−277	−243	4.10	−90	906	1.33

**Table 7:** Mechanical properties of the PBF-LB/M-processed samples.

Sample	Hardness in HV1	Yield Strength Rp0.2 in MPa	Tensile Strength Rm in MPa	Elongation at Fracture A5 in %
A-PBF	183.8 ± 2.6	481.8 ± 20.4	594.4 ± 22.2	51.1 ± 0.3
B-PBF	253.2 ± 8.1	593.3 ± 20.0	758.3 ± 19.1	51.3 ± 0.5
C-PBF	234.0 ± 15.0	527.6 ± 15.08	684.6 ± 12.2	30.0 ± 0.3

**Table 8:** Derived compositional range of 316L type stainless steel for PBF-LB/M processing in mass%.

	C	Si	Mn	Cr	Mo	Ni	N	Fe
min.	-	-	-	17.0	2.00	~10.00	-	bal.
max.	0.03	0.75	2.00	18.0	3.00	~10.00	0.10	bal.

## Data Availability

Data sharing is not applicable to this article.
